# Transition from child-centred to adult-oriented healthcare systems for young people with neurodisability: a scoping review protocol

**DOI:** 10.12688/hrbopenres.13095.1

**Published:** 2020-09-04

**Authors:** Jennifer Fortune, Paul Murphy, Nabil Merchant, Claire Kerr, Thilo Kroll, Aisling Walsh, Meriel Norris, Grace Lavelle, Jennifer Ryan

**Affiliations:** 1 RCSI University of Medicine and Health Sciences, Dublin, Ireland; 2 Queen’s University Belfast, Belfast, Northern Ireland, UK; 3 University College Dublin, Dublin, Ireland; 4 Brunel University London, London, England, UK; 5 King’s College London, London, England, UK

**Keywords:** neurodisability, neurodevelopmental disorders, transition to adult care, transitional care, young adult, adolescent, scoping review

## Abstract

**Background:** The transition from child-centred to adult-oriented healthcare is a challenging time for young people with neurodisability. As the prevalence of neurodisability increases, greater numbers of young people will eventually transfer to the adult healthcare system. While there is a growing recognition of the importance of providing quality, transitional care, little is known about how to manage and optimise this process for young people with neurodisability. The objective of this scoping review is to examine and map existing literature related to the transition from child-centred to adult-oriented healthcare systems for young people with neurodisability.

**Methods:** Systematic literature searches of OVID MEDLINE, EMBASE, PsycINFO, CINAHL, Cochrane Library and Web of Science will be conducted from inception to present. A structured iterative search of grey literature will be conducted. This review will consider all study designs examining the transition from child to adult health services in neurodisability. Two reviewers will independently screen each retrieved title and abstract and assess full-text articles against the inclusion criteria to determine eligibility. Data will be extracted and synthesised quantitatively and qualitatively. The process and reporting will follow PRISMA-ScR guidelines.

**Conclusion:** This review will provide a broad and systematically mapped synthesis of the extent and nature of the available published and unpublished literature on transition from child-centred to adult-oriented healthcare systems in neurodisability. The results will be used to determine gaps in the current evidence base in order to prioritise areas for future research.

## Introduction

Neurodisability describes congenital or acquired long-term conditions that are attributed to impairment of the brain and/or neuromuscular system and create functional limitations ^1^. Neurodisability encompasses conditions characterised by physical, intellectual, behavioural or sensory impairments, such as cerebral palsy, autism spectrum disorders and epilepsy ^2^. The prevalence of neurodisability is increasing ^3^. Individually, many conditions in this heterogeneous group are rare but when grouped together they are common ^4^. Children and young people (CYP) with neurodisability represent between 6 and 9% of the general population ^5– 7^. Advances in medical management and treatment mean many CYP with neurodisability are surviving to need care as adults ^8– 10^.

The transition from adolescence to adulthood is associated with profound physiological, psychological, and social changes ^11^ as young people orientate towards a greater degree of independence in many concurrent areas including relationships, housing, education and employment ^12^. The complexity of this process is amplified for CYP with long-term conditions who are simultaneously negotiating the developmental process of adolescence and independence in managing a long-term condition ^13^. Adaptation from a supported environment to one of individual responsibility and independence in self-advocacy and management during this developmental phase is particularly challenging for individuals with neurodisability who may have communication, physical or learning difficulties, higher rates of comorbid health problems ^1^, and who utilise healthcare services more intensively than their peers ^14, 15^.

The term transition has been adopted to describe the complex and continuous process of preparing CYP with long-term conditions to move from child-centred to adult-oriented healthcare systems ^16^. Transition is therefore distinct from transfer, the latter being considered as a one-time event when the young person moves from a child-centred to adult-oriented healthcare setting ^17^. The transition process aims to maximise potential and lifelong functioning through the provision of cohesive and continuous, developmentally appropriate healthcare as the individual moves to adult health services ^18^. Ideally, this process is coordinated, comprehensive, and patient-centred spanning adolescence to adulthood ^18^, preferably beginning when the individual is 13–14 years of age and continuing until they are capable of taking full responsibility for their health ^19^. However, the transition process is frequently turbulent and experienced negatively by CYP, their families and caregivers ^20^. Obstacles to successfully implementing transition include funding limitations, lack of continuity and coordination between child and adult services ^21– 23^ and limited training opportunities and specialist expertise in the adult system ^24– 27^. In addition, there are significant changes to healthcare provider relationship and reduced support levels in the adult environment ^28^.

Suboptimal transition to adult health care is associated with diminished treatment adherence and interruption or loss to follow-up ^29^. This discontinuity leaves CYP vulnerable to adverse health consequences including functional decrements, medical complications and a heightened risk of hospital admissions ^30– 32^ as well as poor psychological, social and vocational outcomes ^12, 33– 36^. Preventing such declines for CYP with neurodisability is a healthcare priority ^19^. This is reflected in the increasing volume of research examining transition and transfer of care in neurodisability in recent years.

To date, literature has explored effective and efficient features of transition practices and programmes ^37– 40^, explored the perspectives of healthcare professionals ^25, 26^ and experiences of young people and their families ^34, 41– 44^ and the impact of transitional care on measurable outcomes ^45– 47^. A number of scoping reviews have explored transition among CYP with physical ^48^, mental health ^49, 50^ and endocrine, neurological and gastrointestinal conditions ^51, 52^. Evidence from these reviews may not be generalizable to neurodisability due to the range and complexity of these conditions and the greater need for service coordination ^53^. A scoping review protocol by Bogassian and colleagues is the only one available looking specifically at transition and neurodisability ^54^. However, their review focus considers the ethical issues encountered in transition programmes only. Young people with neurodisability constitute a unique and growing population to whom a well-managed and executed transition process can be valuable. Therefore, a comprehensive synthesis of the literature on transition for CYP with neurodisability is needed to consider what is known in order to guide future research and improve transition care.

A scoping review will be undertaken to explore existing literature relating to transition for CYP with neurodisability. This methodology is appropriate as it will provide a comprehensive map of key concepts underpinning the research area and a substantial overview of the types and sources of evidence available in the current body of literature ^55^. It will clarify the aspects of transition which have been the focus of research initiatives to date and identify any knowledge gaps or research deficits that exist within the field that require further research ^55, 56^. 

## Methods

### Design

The methodology for this review draws on the five-stage framework outlined by Arksey and O’Malley ^55^ and more recent refinements to the methodology proposed by Levac *et al.* ^56^ and the Joanna Briggs Institute (JBI) ^57^. The optional sixth stage, ‘consultation with relevant stakeholders’ will not be included as part of this review. This protocol follows the Preferred Reporting Items for Systematic reviews and Meta-Analyses extension for Scoping Reviews (PRISMA- ScR) guidelines ^58^ to ensure rigour in reporting.

### Stage 1: Identifying the research questions

The primary aim of this review is to determine and describe the extent and nature of available evidence addressing transition for CYP with neurodisability and to identify gaps in the existing literature.

Several secondary questions will guide the subsequent stages of the scoping review.

1. What is the current volume and yearly distribution of evidence on transition in CYP with neurodisability?
2. What types of studies on transition in CYP with neurodisability have been conducted (e.g. quantitative, qualitative or mixed-method methodologies)?
3. In which settings and geographical contexts have previous transition studies in neurodisability been conducted?
4. Which conditions have been included in previous studies?
5. How has the concept of ‘transition’ been defined, operationalised and measured in the literature in relation to people with neurodisability?
6. What involvement did CYP and their family/caregivers have in the design, conduct and dissemination of the research?
7. Which theories, models or frameworks have been used to inform transition in neurodisability?

### Inclusion criteria

The inclusion criteria for this scoping review will be guided by the population, concepts and context (PCC) approach ^56^. Inclusion and exclusion criteria are summarised in Table 1.

**Table 1.** Inclusion and exclusion criteria of study selection.

Inclusion	Exclusion
Studies related to transition from child-centred to adult- oriented healthcare systems	Subject of the study is not related to transition from child-centred to adult-oriented healthcare systems
Study sample includes people with neurodisability, families and caregivers of people with neurodisability or health care providers, programme managers and policymakers who work with people with neurodisability	Study sample does not include people with neurodisability, families and caregivers of people with neurodisability or health care providers, programme managers and policymakers who work with people with neurodisability
Studies published in English	Non-English language studies

***Participants*.** A multitude of definitions for neurodisability exist in the literature. Historically the group of conditions encompassed by the term neurodisability were interchangeably referred to as neurodevelopmental disorders ^59^ or neurodevelopmental disabilities ^60^. For uniformity, the term used throughout will be neurodisability. In the context of this review, we will define neurodisability as “A group of congenital or acquired long-term conditions that are attributed to impairment of the brain and/or neuromuscular system and create functional limitations. A specific diagnosis may or may not be identified. Conditions may vary over time, occur alone or in combination, and include a vast range of severity and complexity” ^1^. For the purpose of this review we will include people who experience disturbances of movement, cognition, communication or emotion and behaviour. Studies including male and female CYP with neurodisability will be included. Studies that focus on families, caregivers, health care providers, programme managers and policymakers involved in the transition process will be included.

***Concept*.** The concept examined by this scoping review is the transition from child-centred to adult-orientated healthcare systems. In the context of this review we will define transition as “a purposeful, planned process that addresses the medical, psychosocial and educational/vocational needs of adolescents and young adults with long-term physical and medical conditions as they move from child-centred to adult oriented healthcare systems” ^16^.

***Context*.** This scoping review will consider studies on transition that have been conducted in any setting such as hospitals, healthcare settings, acute care, primary care, special care, home-care or the community. The context will not be limited to specific geographic location.

***Types of evidence sources*.** The review will consider studies of any design that address transition including qualitative, quantitative and mixed-methods methodology. Quantitative studies will include both experimental (e.g., randomised trials, non-randomised trials) and observational (e.g., cohort, cross-sectional) study designs. Case series and individual case reports will also be included. Qualitative studies will include designs such as grounded theory, ethnography, phenomenology, action research and qualitative descriptive. Text and opinion papers will be considered for inclusion if they are published in peer-reviewed journals. In addition, all types of reviews (e.g., systematic reviews, narrative reviews) will be included. Grey literature will also be considered for inclusion in the review.

### Stage 2: Identifying relevant studies

A comprehensive search strategy was developed in consultation with an information specialist. To develop the search strategy, an initial limited search was conducted in OVID MEDLINE and CINAHL to identify articles relevant to the topic area. Key words and index terms were identified from the title and abstract of relevant articles and used to inform the search strategy. Search terms included key words and index terms relating to neurodisability, transition and young people. The search strategy for OVID MEDLINE can be found in the online supplementary material (see *Extended data* ^61^). It will be modified as necessary for the other databases. We will search the following electronic databases from inception to the present date: OVID MEDLINE, EMBASE, PsycINFO, CINAHL, Cochrane Library and Web of Science. We will also perform targeted searches for grey literature through OpenGrey, BASE (Bielefeld Academic Search Engine) and Google. Finally, the literature search will be supplemented by hand searching reference lists of included reports. Only reports published in English will be included. Literature searches will be completed by an information specialist.

### Stage 3: Study selection

All identified citations will be collated and uploaded into EndNote (Clarivate Analytics, PA, USA), and duplicates removed. Two reviewers will independently screen the titles and abstracts of the literature search results considering the eligibility criteria for the review using Rayyan QCRI ^62^. Full texts of potentially eligible studies will be obtained and reviewed by two reviewers independently. Prior to commencing the screening process, two reviewers will conduct a calibration exercise to ensure reliability in correctly screening for inclusion. It will entail independently screening a random sample of the included citations by each reviewer. If low agreement is observed between the reviewers, eligibility criteria will be modified. Discrepancies will be resolved by discussion between reviewers. A third reviewer will be consulted if consensus is not achieved between reviewers.

### Stage 4: Charting the data

Data charting will be conducted using a standardised form, developed from the JBI data extraction tool ^57^. Two reviewers will independently pilot the form on a random sample of included reports. If poor agreement is found, the data extraction form will be revised iteratively and the training exercise will be repeated ^55^. The data charted will include specific details about the population, concept, context, study methods and key findings of significance to the scoping review objective and questions. Authors of papers will be contacted to request missing or additional data, where required. A quality appraisal will not be undertaken in keeping with guidance on scoping review conduct ^63^.

### Stage 5: Collating, summarising and reporting the results

Results of the literature search and study screening process will be presented in a PRISMA-ScR flow diagram ^58^. Charted data will be synthesised quantitatively and qualitatively. For example, summary statistics will be used to describe the current volume, yearly distribution, countries of origin, sample characteristics and methodological design. Key concepts will be summarised using descriptive content analysis. Results will be presented in tabular, graphic or diagrammatical formats according to key findings and knowledge gaps.

### Study status

At the time of publication of this protocol, database searches have been completed.

## Discussion

Given the challenges experienced by CYP with neurodisability during the transition to adult health care, there is an urgent need to better understand this process. This scoping review will broadly and systematically explore what is known about transition in neurodisability. Findings will be used to identify knowledge gaps to direct future research and provide a foundation for developing research priorities. The findings of the review will be published in an open-source journal, presented at national and international conferences, and shared with clinicians, young people and families through organisations for people with disability.

## Data availability

### Underlying data

No underlying data are associated with this article.

### Extended data

Open Science Framework: Transition from child-centred to adult-oriented healthcare systems for young people with neurodisability: a scoping review protocol. https://doi.org/10.17605/OSF.IO/DX8ZF ^61^.

This project contains the following extended data:

- OVID MEDLINE search strategy.pdf
- Draft data charting_data extraction template.pdf
- PRISMA-P checklist.pdf

Extended data are available under the terms of the Creative Commons Zero "No rights reserved" data waiver (CC0 1.0 Public domain dedication).
