# Supplementary material for: Transition from child-centred to adult-oriented healthcare systems for young people with neurodisability: a scoping review protocol
Source: HRB Open Res. 2020 Sep 4;3:61. [Version 1] doi: 10.12688/hrbopenres.13095.1 (PMC7656277; doi:10.12688/hrbopenres.13095.1)
Supplement: Peer review history

# Peer review: Reviewer response for version 1

**DOI:** 10.21956/hrbopenres.14196.r28291

**Published:** 2020-11-30

**Authors:** Katherine Langdon

**Affiliations:** 1 Department of Paediatric Rehabilitation, Perth Children's Hospital, Nedlands, WA, Australia

Thanks for the opportunity to comment. I wonder what issues you will find in the search to pin down the research question? There are many unique circumstances that ought to be addressed. There is variability between the neurodisabilities in the health related comorbidities and the earlier these occur, the better they are described and the more dire the outcomes in the event of poor management, the more likely that these conditions will have a transition process in place e.g. spina bifida. Where serious health related problems occur later in the trajectory of a condition e.g. cerebral palsy, the more lacklustre the transition program in my experience. 

 Success or otherwise is also dependent on the agency of the child which is a subject I have not seen much research about in the disability literature.  

 Children in state care are a unique problem in transition programs as medical transition can be accompanied by a transition in housing and guardianship. 

 The adult perspective of graduates of childhood conditions is also unpredictable and influences the willingness of adult engagement. Severe disability can be met with the automatic presumption of "palliation" and with it an assumption that death is "imminent" despite the child already having lived for 17 or so years, once services are sought in the adult sector and levels of care can be set on the basis of these value judgments.  While it may not come into the scope of your questions, I wonder too whether CYP with congenital disabilities are not differentiated from adults with acquired disabilities who have lost skills they once had. I wonder whether this too affects the interest level in adult engagement with paediatric neurodisability graduates. There is a need to look into these clinically relevant scenarios witnessed in practice but not (yet?) in the literature.

Is the study design appropriate for the research question?

Yes

Is the rationale for, and objectives of, the study clearly described?

Yes

Are sufficient details of the methods provided to allow replication by others?

Yes

Are the datasets clearly presented in a useable and accessible format?

Not applicable

Reviewer Expertise:

paediatric cerebral palsy, spinal cord pathology, acquired brain injury

I confirm that I have read this submission and believe that I have an appropriate level of expertise to confirm that it is of an acceptable scientific standard.

# Peer review: Reviewer response for version 1

**DOI:** 10.21956/hrbopenres.14196.r27934

**Published:** 2020-11-10

**Authors:** Divya Jindal-Snape

**Affiliations:** 1 Transformative Change: Education and Life Transitions (TCELT) Research Centre, School of Education and Social Work, University of Dundee, Dundee, UK

This article presents the protocol for a scoping review that will map previous literature on transitions of children and young people with neurodisability as they move from child- to adult-centred healthcare systems. This includes investigating the key concepts, and nature and type of evidence presented in this literature.

 This is an important area of research. The protocol is clearly structured with explicit and transparent methodology. Most transitions researchers do not make the conceptualization and operationalisation of transitions explicit. Therefore, this aspect adds strength to the proposed scoping review.

 It is important, however, to focus on all multiple transitions they will be experiencing at the same time and try to explore what other transitions have been (potentially implicitly) presented in the health literature you currently plan to review. These could, for instance, be developmental, educational, relationship transitions. This is important as their transitions to healthcare systems will trigger, and be triggered by, other transitions. This will also be the case for the families and professionals, with their transitions interacting with those of children and young people.

 Although I understand this is outside the scope of the proposed review, I believe it will be useful for the authors to review conceptualisation and theoretical frameworks of transitions in other disciplines too. Also, it will be important to publish your scoping review beyond journals from a particular discipline as there is a gap in this area.

Is the study design appropriate for the research question?

Yes

Is the rationale for, and objectives of, the study clearly described?

Yes

Are sufficient details of the methods provided to allow replication by others?

Yes

Are the datasets clearly presented in a useable and accessible format?

Yes

Reviewer Expertise:

Education, health education, educational and life transitions, systematic literature review, creativity, inclusion, voice

I confirm that I have read this submission and believe that I have an appropriate level of expertise to confirm that it is of an acceptable scientific standard.

# Peer review: Reviewer response for version 1

**DOI:** 10.21956/hrbopenres.14196.r27932

**Published:** 2020-10-06

**Authors:** Tim Rapley

**Affiliations:** 1 Department of Social Work, Education and Community Wellbeing, Northumbria University, Newcastle upon Tyne, UK

This protocol outlines a scoping review on transition from child to adult healthcare services and systems for young people with neurodisability. 

 It is clear, accessible and well written and outlines the process in enough detail. The primary research question is timely as are the secondary focus (especially how transition is operationalized)

Is the study design appropriate for the research question?

Yes

Is the rationale for, and objectives of, the study clearly described?

Yes

Are sufficient details of the methods provided to allow replication by others?

Yes

Are the datasets clearly presented in a useable and accessible format?

Not applicable

Reviewer Expertise:

Health Services Research, Implementation, CYP

I confirm that I have read this submission and believe that I have an appropriate level of expertise to confirm that it is of an acceptable scientific standard.

# Response

**Published:** 2020-10-19

**Authors:** Jennifer Fortune

**Affiliations:** RCSI, Ireland

Thank you for reviewing our protocol. We appreciate your comments, particularly your feedback on our research question and secondary focus.
